# Ankle Joint Angle Influences Relative Torque Fluctuation during Isometric Plantar Flexion

**DOI:** 10.3390/bioengineering10030373

**Published:** 2023-03-18

**Authors:** Fandi Shi, William Zev Rymer, Jongsang Son

**Affiliations:** 1 Department of Biomedical Engineering, McCormick School of Engineering, Northwestern University, Evanston, IL 60208, USA; 2 Shirley Ryan AbilityLab (Formerly the Rehabilitation Institute of Chicago), Chicago, IL 60611, USA; 3 Department of Physical Medicine and Rehabilitation, Feinberg School of Medicine, Northwestern University, Chicago, IL 60611, USA; 4 Department of Biomedical Engineering, Newark College of Engineering, New Jersey Institute of Technology, Newark, NJ 07102, USA

**Keywords:** force variability, low-frequency oscillation, signal-dependent noise, muscle length

## Abstract

The purpose of this study was to investigate the influence of changes in muscle length on the torque fluctuations and on related oscillations in muscle activity during voluntary isometric contractions of ankle plantar flexor muscles. Eleven healthy individuals were asked to perform voluntary isometric contractions of ankle muscles at five different contraction intensities from 10% to 70% of maximum voluntary isometric contraction (MVIC) and at three different muscle lengths, implemented by changing the ankle joint angle (plantar flexion of 26°-shorter muscle length; plantar flexion of 10°-neutral muscle length; dorsiflexion of 3°-longer muscle length). Surface electromyogram (EMG) signals were recorded from the skin surface over the triceps surae muscles, and rectified-and-smoothed EMG (rsEMG) were estimated to assess the oscillations in muscle activity. The absolute torque fluctuations (quantified by the standard deviation) were significantly higher during moderate-to-high contractions at the longer muscle length. Absolute torque fluctuations were found to be a linear function of torque output regardless of muscle length. In contrast, the relative torque fluctuations (quantified by the coefficient of variation) were higher at the shorter muscle length. However, both absolute and relative oscillations in muscle activities remained relatively consistent at different ankle joint angles for all plantar flexors. These findings suggest that the torque steadiness may be affected by not only muscle activities, but also by muscle length-dependent mechanical properties. This study provides more insights that muscle mechanics should be considered when explaining the steadiness in force output.

## 1. Introduction

Force (or torque) fluctuations in the low-frequency (<1 Hz) band are well-known to arise during voluntary sustained isometric contractions in human skeletal muscles [1]. Such fluctuations, often quantified by standard deviation (SD) or coefficient of variation (CoV), appear to be a useful biomarker to help us understand motor performance. For example, greater force fluctuations have been observed in individuals in aging [2,3,4], musculoskeletal injuries [5,6] and neurophysiology disorders [3], demonstrating significant correlation with motor function [1,7]. Accordingly, a systematic investigation into underlying mechanisms of force fluctuations is essential for understanding motor activities and for improving motor control ability in broad clinical populations.

Force fluctuations were found to be closely related to variability in the recruitment and rate-coding properties of the motor units [8]. Both experimental and simulation studies demonstrated a linear increase of SD for force (F_SD_) with increasing voluntary isometric contraction level [9]. Such increase in F_SD_ can be explained by the presence of signal-dependent noise that appears to be associated with the statistical distribution of the motoneuron spike trains [10]. Previous studies have also suggested that CoV for force (F_CoV_) decreases non-linearly as the contraction level increases by up to approximately 20% of maximum voluntary isometric contraction (MVIC) [9]. Such reduction in F_CoV_ can be explained, in part, by the characteristics of motor unit recruitment and motor unit discharge variability [7,11]. For instance, significant correlations between F_CoV_ and CoV for motor unit discharge rate were found in younger individuals [7]. Collectively, low-frequency oscillations in force may be consequences of variability in a single motor unit’s firing rate or low-frequency oscillations in the neural drive to the muscle, commonly estimated by the cumulative spike trains or the rectified-and-smoothed EMG (rsEMG) [12,13,14].

Until now, many studies have suggested the role and significance of neurophysiological factors in the generation of force fluctuation but lacked the materials to address how muscle mechanics can modulate the force fluctuation. Considering muscle length-mediated changes in contractile properties (e.g., time-to-peak, half-relaxation time, or force-length characteristics) [15] as well as in afferent feedback [16], it is likely that muscle mechanics can also impact the steadiness of the force output. A few studies using the joint angle as a proxy for muscle length showed that shorter muscle length led to an increase in the plantar flexion torque variability, when compared with longer muscle length at the same force level [17,18]. This may be due to the finding that the twitch time of the recruited motor units are shortened at shorter muscle lengths [15], and more motor units are recruited [19]. Both factors may induce a decreased firing rate or larger variability in firing rate and thus lead to higher force fluctuation. Moreover, any changes in muscle length can result in changes in spinal cord excitability. At a more flexed ankle joint angle, there is less stretch on muscle spindles (Ia afferent) and thus reduced excitatory drive to the motoneuron pool, potentially affecting the steadiness of motor output [20].

The purpose of this study was to evaluate the influence of changes in muscle length on the plantar flexion torque variability and oscillations in muscle activities during submaximal voluntary isometric contractions in neurologically intact individuals. To assess the oscillations in muscle activities, we collected the surface EMG signals and obtained the SD for the rsEMG of triceps surae muscles. We hypothesized that torque variability will be affected by muscle length, presumably related to underlying changes in low-frequency oscillations in neural drive and/or muscle mechanics.

## 2. Materials and Methods

### 2.1. Participants

Eleven young healthy individuals (Age: 27 ± 1.5 yrs.; F/M: 5/6) participated in this study after providing consent. All procedures were approved by the Institutional Review Board in Northwestern University and complied with the Helsinki declaration.

### 2.2. Experimental Setup

**Figure 1:**
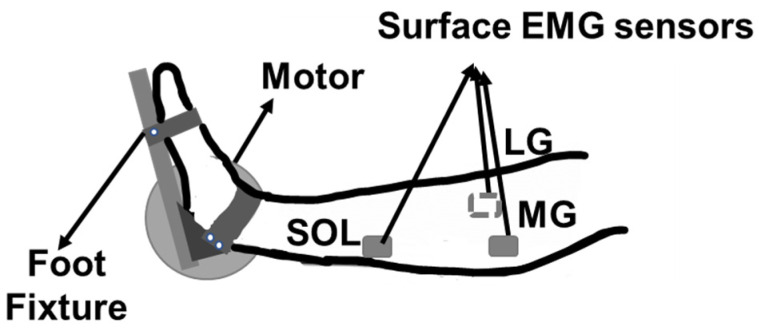
Schematic diagram of experimental setup.

Participants were asked to sit upright in a Biodex chair with the knee fully extended (Figure 1). The right foot was fixed onto a footplate attached to a 6-axis loadcell (Omega160, ATI Industrial Automation, Apex, NC, USA), secured via an adhesive foot strap. Single differential surface EMG electrodes (Bagnoli, Delsys Inc., Boston, MA, USA) were placed on the triceps surae muscles (i.e., middle part of the lateral gastrocnemius (LG), soleus (SOL), and tibialis anterior, and on the distal part of the MG muscle belly) with a ground electrode attached on the bony landmark (i.e., lateral malleolus). The locations of electrodes were determined after muscle palpations on the muscles of interest. Prior to electrode placements, the sites were cleaned with alcohol pads. EMG and plantar flexion torque signals were performed simultaneously at a sampling frequency of 2 kHz (NI USB-6259 BNC, National Instrument, Austin, TX, USA).

### 2.3. Experimental Protocol

Plantar flexion contractions with the right leg were performed at three ankle joint angles: ankle joint angles at 87°, at 100°, and at 116° (the angle between shank and sole of foot), corresponding to longer, neutral, and shorter muscle lengths, respectively. Each configuration was set by adjusting the foot-plate position. At each ankle joint angle, three MVICs were performed, and the averaged value of these was used to estimate the target submaximal contraction torque. Each subject was asked to perform isometric contractions at 10%, 20%, 30%, 50%, and 70% MVIC. Visual feedback was provided in real time involving reaching the target level from rest state (5–10 s) and then maintaining at the target level ± 3% MVIC for at least 6 s. Each contraction level was repeated three times. To minimize the effects from systematic muscle fatigue, the orders of the ankle joint angles and target contraction levels were randomized, and at least a 30 s break was provided between trials.

### 2.4. Data Analysis

The torque signals were low-pass filtered using a fourth-order Butterworth filter with a cutoff frequency at 6 Hz. Raw EMG signals were band-pass filtered using a fourth-order Butterworth filter with a passband of 20–450 Hz. The filtered EMG signals were then rectified and smoothed by applying a fourth-order Butterworth filter with a cutoff frequency of 2 Hz (hereafter called rsEMG).

**Figure 2:**
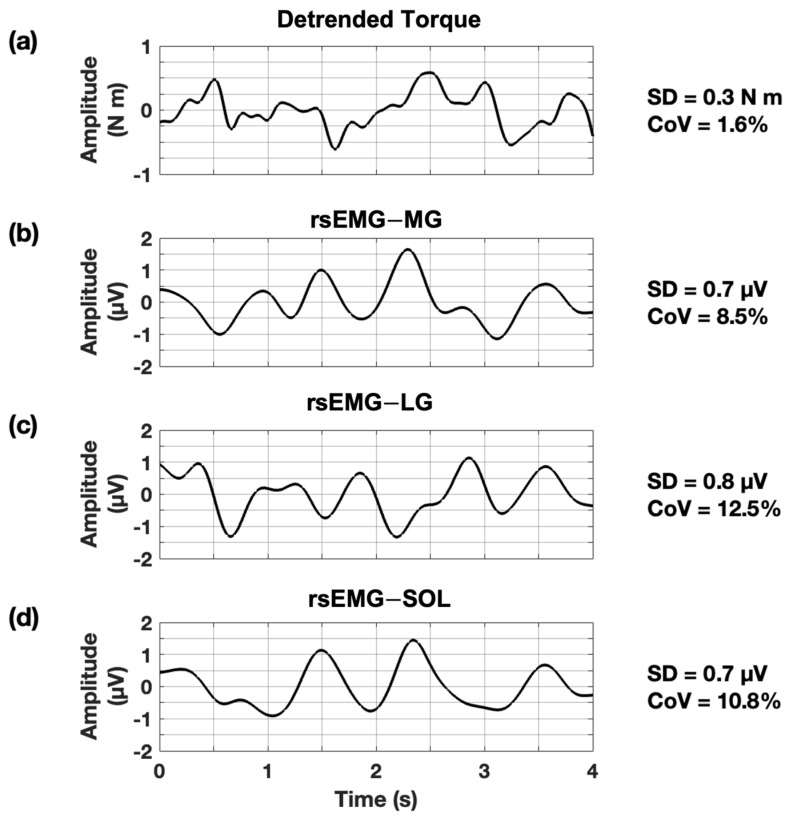
(**a**) Typical force trials of 30%MVIC for 4 s at longer muscle length from a representative subject. (**b**) From the same segment in (**a**), rsEMG from MG muscle; (**c**) rsEMG from LG muscle; (**d**) rsEMG from SOL muscle. All are detrended signals.

To determine a representative stable measure of the variability of muscle contractions for each trial, a 4 s segment in the middle of each sustained contraction was chosen by calculating the minimum standard deviation in the filtered torque signals. Using this time segment, both absolute and relative variability for the filtered torque signals were quantified as SD and as the CoV, respectively. Hereafter, absolute and relative variability for torque is called T_SD_ and T_CoV_. CoV was defined as the ratio of SD to the mean torque value of the segment. Both absolute and relative variability values for the rsEMG of each plantar flexor (MG_SD_, LG_SD_, and SOL_SD_; MG_CoV_, LG_CoV_, and SOL_CoV_) were calculated to examine the oscillations in muscle activities. Figure 2 shows representative detrended torque trials and rsEMG signals from a representative subject.

### 2.5. Statistical Analysis

A Kolmogorov–Smirnov test was performed to assess the normality of the data for each test. Since the normality test on the major outcomes rejected the null hypothesis, a log transformation was applied. Given the evaluation of their skewness and kurtosis after log transformation, the data distributions were acceptable to conduct recommended parametric tests [21]. Thus, a two-way, repeated-measures ANOVA was performed to examine the differences in the log-transformed torque measurement outcomes (T_SD_ and T_CoV_) and oscillations of muscle activities at the five contraction levels and three ankle joint angles via the SPSS (IBM Corp., Armonk, NY, USA) with a significance level (α) of 0.05. When necessary, post hoc pairwise multiple comparisons with Bonferroni correction were used. In any case the sphericity assumption was violated, Greenhouse–Geisser would be used instead.

Stepwise regression analysis was applied to test what is the more dominant contributor to the changes in T_CoV_ with contraction levels and ankle joint angles. To determine the dominant muscle that contributes to the changes in torque variability with contraction levels, the data were collapsed across the contraction levels, and the stepwise regression analysis was conducted at each ankle joint angle. When determining the dominant muscle that contributes to the changes in torque variability with the ankle joint angle at each contraction level, the data were collapsed across the ankle joint angles, and the analysis was conducted at each contraction level.

## 3. Results

### 3.1. Absolute Variability for Torque

**Figure 3:**
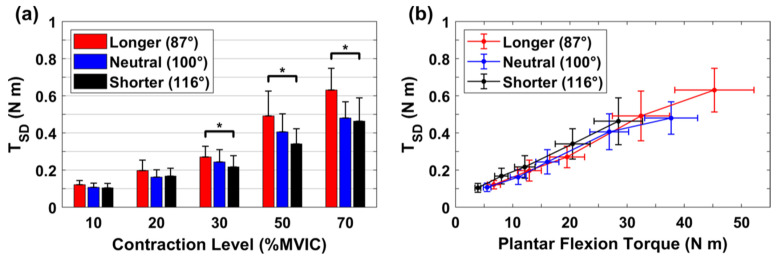
(**a**) Absolute torque variability at different contraction intensities and ankle joint positions. (**b**) Relationships between absolute torque variability and actual plantar flexion torque at different ankle joint positions. Asterisk indicates a significant difference (* *p* < 0.050) between the longer muscle length and the shorter one. T_SD_ indicates the absolute torque variability. Error bar indicates SEM.

The ANOVA analysis revealed significant main effects from both the contraction level (F (4, 40) = 142.458, *p* < 0.001) and ankle angle (F (2, 20) = 6.547, *p* = 0.006) on T_SD_. No significant interaction was also found between the contraction level and ankle angle (F (8, 80) = 1.325, *p* = 0.243). As shown in Figure 3a, further analysis suggested that T_SD_ at the longer muscle length was observed to be significantly larger than that at the shorter muscle length when the contraction level is at 30% (*d_z_* = 0.274, *p* = 0.026), 50% (*d_z_* = 0.420, *p* = 0.029) and 70% MVIC (*d_z_* = 0.414, *p* = 0.006). As shown in Figure 3b, T_SD_ increases linearly with the actual plantar flexion torque regardless of muscle length.

### 3.2. Absolute Variability for rsEMG

The average MG_SD_, LG_SD_, and SOL_SD_ at different contraction levels and ankle angles are presented in Table 1. Significant effects of contraction level are indicated for all three muscles (MG: F (4, 40) = 240.271, *p* < 0.001; LG: F (4, 40) = 145.711, *p* < 0.001; and SOL: F (4, 40) = 72.440, *p* < 0.001). For the MG muscle, the ANOVA results indicated that there is no significant effect of the ankle angle (F (2, 20) = 1.240, *p* = 0.311) or no interaction (F (8, 80) = 0.589, *p* = 0.784). Similarly, LG_SD_ was not affected by the ankle angle (F (2, 20) = 0.612, *p* = 0.552) or the interaction (F (8, 80) = 0.598, *p* = 0.777). For the SOL muscle, there are no significant effects from both the ankle angle (F (2, 20) = 1.168, *p* = 0.331) and the interaction (F (8, 80) = 1.101, *p* = 0.372).

### 3.3. Relative Variability for Torque

**Figure 4:**
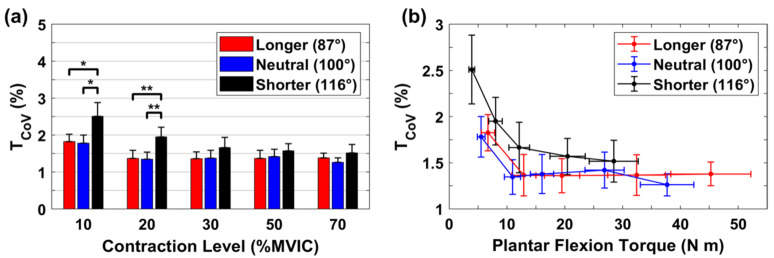
(**a**) Relative torque variability at different contraction levels and joint angles. (**b**) Relative torque variability at different plantar flexion torque and joint angles. Asterisk indicates a significant difference (* *p* < 0.050, ** *p* < 0.001) detected between different muscle lengths. T_CoV_ indicates the relative torque variability. Error bars indicate SEM.

The statistical analysis suggested a significant main effect from the contraction level (F (4, 40) = 8.037, *p* < 0.001) and ankle joint angle (F (2, 20) = 11.177, *p* < 0.001) on T_CoV_, but there was no significant effect from the interaction between the contraction level and ankle joint angle (F (8, 80) = 1.536, *p* = 0.158). As shown in Figure 4a, T_CoV_ at the ankle joint angle of 116° was significantly higher than that at the other two ankle joint angles at the contraction of 10% MVIC (Shorter vs. Longer: *d_z_* = 0.724, *p* = 0.031; Shorter vs. Neutral: *d_z_* = 0.743, *p* = 0.039) and 20% MVIC (Shorter vs. Longer: *d_z_* = 0.734, *p* < 0.001; Shorter vs. Neutral: *d_z_* = 0.818, *p* < 0.001). As shown in Figure 4b, T_CoV_ at ankle joint angle of 116° tends to be the highest at the same plantar flexion torque compared with the other two ankle joint angles.

### 3.4. Relative Variability for rsEMG

The average MG_CoV_, LG_CoV_, and SOL_CoV_ at each ankle angle are presented in Table 2. For the MG muscle, the ANOVA results indicated that there is no significant effect from the ankle joint angle (F (2, 20) = 1.529, *p* = 0.241) and no significant interaction between the ankle joint angle and the contraction level (F (8, 80) = 0.463, *p* = 0.879). Although it shows a significant dependence on the contraction level (F (4, 40) = 5.311, *p* = 0.002), it only shows a significant reduction from 10% to 50% MVIC (*p* = 0.036) at the longest muscle length and then remained relatively constant among other comparisons. For the LG muscle, no significant dependence was observed from the contraction level (F (4, 40) = 0.822, *p* = 0.519), ankle joint angle (F (2, 20) = 0.572, *p* = 0.573), or their interaction (F (3.788, 37.872) = 1.065, *p* = 0.396). For the SOL muscle, there is no significant effect from the contraction level (F (4, 40) = 1.434, *p* = 0.240) and the interaction between the ankle joint angle and the contraction level (F (3.413, 34.125) = 0.419 *p* = 0.765). It shows a significant effect from the ankle joint angle (F (2, 20) = 5.915, *p* = 0.010) with a significant reduction in SOL_CoV_ at 20% and 70% MVIC when the ankle angle is changed from 116° to 87°, and a reduction at 30% MVIC when it is changed from 100° to 87°.

### 3.5. Contributions from Relative Variability of rsEMG to the Resultant Relative Torque Variability

It appears that individual muscle contributions to T_CoV_ may be different at different ankle joint angles (Table 3). The stepwise regression model revealed that the MG_CoV_ contribution to T_CoV_ was significant at all tested ankle angles. The LG_CoV_ significantly contributed to T_CoV_ at both the neutral and shorter muscle lengths, whereas the SOL_CoV_ contribution was significant only at the longer muscle length.

When considering the individual muscle contributions to T_CoV_ at different contraction intensities (Table 4), MG_CoV_ seems to be a more dominant contributor compared to the other two muscles, as supported by its significant contribution to T_CoV_ from 20 to 70% MVIC. The contribution of both LG_CoV_ and SOL_CoV_ to T_CoV_ was significant only at 30 and 50% MVIC. Interestingly, T_CoV_ at 10% MVIC was not explained significantly by any of triceps surae.

## 4. Discussion

The main findings of this study are as follows: (1) absolute torque variability (T_SD_) increased linearly with increasing the contraction level, and a significantly higher T_SD_ was observed during moderate-to-high submaximal contractions (i.e., 30–70% MVIC) at the longer muscle length compared to the shorter muscle length, while the oscillations in muscle activities (absolute variability for rsEMG of each muscle—MG_SD_, LG_SD_, and SOL_SD_) remained relatively constant at different ankle joint angles; (2) T_SD_ was found to be linearly increased with actual torque output regardless of ankle joint angles; (3) relative torque variability (T_CoV_) was found to be significantly higher during low submaximal contractions (i.e., 10–20% MVIC) at the shorter muscle length compared to the other two muscle lengths, but it appears that there is no significant effect of ankle joint angle on T_CoV_ during moderate-to-high submaximal contractions; and (4) the stepwise regression models suggested that among triceps surae, the MG may play an important role to control the steadiness of plantar flexion torque.

T_SD_ increased monotonically with contraction level at each ankle joint angle, as supported by previous findings [22]. This linear increment with contraction level of T_SD_ may be explained by the presence of the signal-dependent noise (SDN) model, which assumes that there is noise from the motor command and that the amount of noise scales with the magnitude of motor command [10]. Our study further emphasized that the presence of SDN may also hold in a joint with multiple synergists. Studies have demonstrated that T_SD_ is highly correlated with the low-frequency oscillations in the common neural drive, which can be quantified via cumulative spike trains or rsEMG of the target muscles [12,13,14]. This assertion can also be supported by our findings that MG_SD_, LG_SD_, and SOL_SD_ increase monotonically with the contraction level (Table 1).

The significantly larger T_SD_ was found at the longest muscle length during the moderate-to-high contractions (i.e., 30–70% MVIC). Since there are no significant effects from the ankle joint angle on the oscillations in muscle activities (Table 1), it suggests that the significant changes in T_SD_ at the longer muscle length is not likely affected by oscillations in common neural drive; instead, T_SD_ appears to be a linear function of actual torque output (Figure 3b), which further implies that the increment in T_SD_ at the longer muscle during moderate-to-high contractions may result from the higher torque output linked to the length-tension function [23,24,25], while the lack of significant differences in T_SD_ at lower contractions may be due to the fact that the actual torque output in that range across the muscle lengths is similar. This may suggest that absolute torque variability is a function of actual torque output regardless of muscle mechanics.

Our results revealed that at all ankle joint angles, T_CoV_ decreased from a higher value and remained constant afterwards with significance detected at both the neutral (100°) and shorter muscle length (116°), in agreement with previous findings [9]. There are no clear underlying mechanisms for this yet, but it is plausible that such nonlinear relationships can be explained, in part, by motor unit mechanics (i.e., firing patterns and contractile properties), as supported by the previous finding, which demonstrated a significant correlation between CoV for motor unit discharge rate and T_CoV_ at low contraction intensities of the first dorsal interosseous muscles in older individuals [7]. Given that the influence of variability in discharge rate of a single motor unit is almost attenuated by convolution of motoneuron spike trains with motor unit twitches and summation of twitch forces, motor unit discharge variability may affect the steadiness in force output only at low contraction levels. At higher force levels thereafter, the more fused contractions due to more active motor units and their increased firing rates would result in higher absolute but lower relative force variability. However, rsEMG has limitations to provide information regarding an individual MU firing characteristics. To better understand the association between the firing characteristics and force variability, further studies are needed.

T_CoV_ at low contraction levels (i.e., 10–20% MVIC) was significantly higher at the shorter muscle length (Figure 4a). Indeed, to generate the comparable forces at the shorter muscle length, it is most likely that more active motor units (MUs) are recruited and/or higher firing rates of the active MUs are required [19], leading to a more fused status and thus a smaller T_CoV_. The potential underlying mechanisms may involve the changes in twitch properties of the activated MUs (i.e., decreased half-relaxation time) but with a relatively constant firing rate at the shorter muscle length [15], which may lead to less fused status and thus a higher T_CoV_. Another possible mechanism may be related to the inhibited transmission of the low-frequency oscillations in force signals at the shorter muscle length while the muscle is under high slack (i.e., 10–20% MVIC). The lack of significance in the changes of T_CoV_ at moderate-to-high contractions (i.e., 30–70% MVIC in Figure 4a) may be due to the fact that muscle slack uptake occurs while the muscle is with high active tension and thus can limit the effects from changes in muscle length at moderate-to-high contractions. As demonstrated in Table 3, the lack of contribution from any of triceps surae on T_CoV_ at 10% MVIC may further suggest that the relative torque variability at low contractions can be highly affected by muscle mechanics instead of muscle activation. Future studies are required to better understand the potential effects of length-dependent mechanisms on force variability, which have received less attention so far.

Some animal studies demonstrated direct evidence on selective recruitment of different types of motor units, particularly in synergistic muscles [26]. It is then possible that SOL may be recruited first and contribute predominantly to ankle torque generation, thus affecting the changes in torque variability at low contraction levels. However, different from animal models, MG is a mixed muscle containing at least 40% slow-twitch fibers in humans [27]. Our results (Table 3 and Table 4) suggested that MG muscle may play an important role to control the steadiness of plantar flexion torque among triceps surae. Moreover, T_CoV_ at low contraction levels (i.e., 10% MVIC) among different ankle angles was not explained significantly by any of triceps surae, which may provide indirect evidence that torque variability can be highly affected by muscle mechanics instead of by muscle activation strategy at a low contraction level, and thus the potential effects from the recruitment order among triceps surae on the different torque variabilities at different ankle angles may be limited.

Several studies indicated that most of the variability in the force signal during the steady contractions can be explained by fluctuations in the common modulation of the motor unit discharge rate, with low-frequency oscillations over time, indicated by rsEMG [13,28]. It has been proposed to use force variability as a potential measurement approach to evaluate motor function in clinical populations (i.e., stroke survivors) [13,28]. Our study suggested that not only the potential changes in motor function, but also the changes in muscle mechanics, can impact the changes in force variability. Considering that muscle mechanics disorders (i.e., muscle stiffness) can also be highly impacted in individuals with neurological disorders, care should be taken when explaining the changes in force steadiness in clinical populations. Further studies would be needed to understand the relative contribution from changes in muscle mechanics and motor function to the steadiness of the resultant force output.

There are several limitations in our study. Previous research has demonstrated that tibialis anterior (TA) muscle contracts simultaneously [29,30] to maintain the joint stability during isometric contractions. However, our further evaluation on the activation status of the TA muscle shows that the RMS EMG of TA was significantly smaller when compared with MG and LG; moreover, its amplitude was less than 4% of the RMS EMG of TA collected during maximum dorsiflexion, suggesting that the effects from the antagonist on the plantar flexion torque variability may be negligible. We also have limitations on the manipulations of changes in the ankle joint angle due to the constraints of our setup. Our results only demonstrated a significant difference in T_CoV_ between the shorter and the longer/neutral muscle length but lack an observable difference when comparing between the neutral and longer muscle length. This may be due to the small interval of changes in the ankle joint angles. Another limitation is that the targeted population in this study only involves young individuals. Considering that aging and neurological impairment (i.e., stroke survivors) also affect the steadiness of force output [2,3,31] as well as mechanical properties of the muscle–tendon unit [32], it would be interesting to investigate the relative contribution of changes in the mechanical properties to the force variability in different populations. Lastly, it is possible that the higher T_CoV_ observed at the shorter muscle length during lower contractions (10–20% MVIC) can be affected by the smaller torque output at a shorter muscle length. However, considering that the average torque value during 20% MVIC at a longer muscle length (12.9 N m) is comparable to that during 30% MVIC at a shorter muscle length (12.1 N m), our further evaluation suggested that T_CoV_ at the shorter muscle length was ~20% greater compared to the longer muscle length. T_CoV_–torque relation also illustrated that T_CoV_ at the shorter muscle length is likely higher at a given, comparable torque output (Figure 4b). Future studies are necessary to examine torque fluctuations at the matched actual torque.

## 5. Conclusions

This study demonstrated that the absolute torque variability increased during modest submaximal contractions at a longer muscle length, whereas the relative torque variability increased during low submaximal contractions at a shorter muscle length. These findings suggest that the torque steadiness may be affected by both neural drive and muscle mechanics. Future studies are needed to better describe how these neuromuscular properties can influence the variability of force output.

## Figures and Tables

**Table 1:** Absolute variability of MG, LG, and SOL (MG_SD_, LG_SD_, and SOL_SD_) at different contraction intensities and ankle joint angles. L: Longer muscle length (87°); N: Neutral muscle length (100°); S: Shorter muscle length (116°). Data shows as Mean ± SEM. Unit: µV.

Muscle	Position	10% MVIC	20% MVIC	30% MVIC	50% MVIC	70% MVIC
MG	L	0.62 ± 0.13	0.85 ± 0.17	1.18 ± 0.21	1.99 ± 0.40	2.56 ± 0.51
	N	0.52 ± 0.09	0.87 ± 0.16	1.16 ± 0.24	2.04 ± 0.49	2.78 ± 0.64
	S	0.69 ± 0.12	0.98 ± 0.19	1.31 ± 0.27	2.10 ± 0.40	3.18 ± 0.70
LG	L	0.42 ± 0.05	0.65 ± 0.07	0.94 ± 0.10	1.67 ± 0.27	2.37 ± 0.40
	N	0.48 ± 0.09	0.59 ± 0.09	1.06 ± 0.24	1.52 ± 0.27	2.23 ± 0.39
	S	0.49 ± 0.08	0.69 ± 0.11	1.06 ± 0.20	1.75 ± 0.31	2.84 ± 0.46
SOL	L	0.30 ± 0.03	0.30 ± 0.05	0.50 ± 0.06	0.78 ± 0.11	1.01 ± 0.14
	N	0.35 ± 0.06	0.35 ± 0.08	0.57 ± 0.13	0.70 ± 0.14	0.84 ± 0.13
	S	0.31 ± 0.06	0.31 ± 0.05	0.48 ± 0.10	0.69 ± 0.14	1.02 ± 0.19

**Table 2:** Relative variability of MG, LG, and SOL (MG_CoV_, LG_CoV_,and SOL_CoV_) at different contraction intensities and ankle joint angles. L: Longer muscle length (87°); N: Neutral muscle length (100°); S: Shorter muscle length (116°). Data shows as Mean ± SEM. Unit: %.

Muscle	Position	10% MVIC	20% MVIC	30% MVIC	50% MVIC	70% MVIC
MG	L	9.98 ± 0.68	8.73 ± 0.50	8.41 ± 0.62	8.74 ± 0.62	8.25 ± 0.55
	N	8.81 ± 0.70	7.77 ± 0.52	7.52 ± 0.43	8.07 ± 0.61	8.13 ± 0.62
	S	8.66 ± 9.68	8.03 ± 0.50	7.60 ± 0.67	8.28 ± 0.56	8.42 ± 0.55
LG	L	8.30 ± 0.37	8.01 ± 0.72	8.04 ± 0.41	8.41 ± 0.76	8.77 ± 0.96
	N	8.17 ± 0.14	7.77 ± 0.61	8.46 ± 0.80	7.47 ± 0.62	8.05 ± 0.68
	S	8.19 ± 0.48	7.46 ± 0.51	7.64 ± 0.51	8.16 ± 0.38	8.41 ± 0.53
SOL	L	7.53 ± 0.40	6.76 ± 0.35	6.98 ± 0.33	7.31 ± 0.46	7.38 ± 0.31
	N	7.78 ± 0.36	7.46 ± 0.47	7.08 ± 0.36	7.36 ± 0.67	8.83 ± 1.17
	S	9.02 ± 0.93	8.30 ± 0.37	8.63 ± 0.58	8.80 ± 0.82	9.29 ± 0.68

**Table 3:** Stepwise regression model to identify possible predictors of the relative variability for torque (T_CoV_) out of the relative variabilities for EMG burst (MG_CoV_, LG_CoV_, and SOL_CoV_) across the contraction levels at each ankle joint angle. Data are shown as the estimated coefficient ±95% confidence interval. The significant predictors are in boldface type and the most important variable is highlighted. * *p* < 0.05. ** *p* < 0.001.

	MG	LG	SOL
**Longer**	* 0.14 ± 0.08	0.04 ± 0.07	* 0.18 ± 0.13
**Neutral**	* 0.08 ± 0.07	** 0.13 ± 0.07	0.04 ± 0.06
**Shorter**	* 0.19 ± 0.11	* 0.18 ± 0.14	−0.01 ± 0.09

**Table 4:** Stepwise regression model to identify possible predictors of the relative variability for torque (T_CoV_) out of the relative variabilities for EMG burst (MG_CoV_, LG_CoV_, and SOL_CoV_) across the ankle joint angles at each contraction level. Data are shown as the estimated coefficient ±95% confidence interval. The significant predictors are in boldface type and the most important variable is highlighted. * *p* < 0.05. ** *p* < 0.001.

% MVIC	MG	LG	SOL
**10**	0.03 ± 0.15	0.17 ± 0.28	0.04 ± 0.16
**20**	* 0.22 ± 0.14	0.04 ± 0.13	0.08 ± 0.16
**30**	** 0.19 ± 0.08	* 0.15 ± 0.09	* 0.12 ± 0.10
**50**	* 0.10 ± 0.09	* 0.11 ± 0.09	* 0.11 ± 0.08
**70**	** 0.19 ± 0.10	0.04 ± 0.09	0.03 ± 0.07

## Data Availability

The data that support the findings of this study are available from the corresponding author upon reasonable request.
